# Ginkgo biloba: An adjuvant therapy for progressive normal and high tension glaucoma

**Published:** 2012-02-09

**Authors:** A.K. Cybulska-Heinrich, M. Mozaffarieh, J. Flammer

**Affiliations:** Department of Ophthalmology, University of Basel, Basel, Switzerland

## Abstract

Gingko biloba has been used for hundreds of years to treat various disorders such as asthma, vertigo, fatigue and, tinnitus or circulatory problems. Two of the main extracts are EGb761 and LI 1370. Most pharmacological, toxicological and clinical studies have focused on the neuroprotective value of these two main extracts. Neuroprotection is a rapidly expanding area of research. This area is of particular interest due to the fact that it represents a new avenue of therapy for a frustrating disease that may progress despite optimal treatment. One such disease is glaucoma.

Glaucoma leads to the loss of retinal ganglion cells and their axons but also to tissue remodelling which involves both the optic nerve head and the retina. In the retina the astrocytes get activated. In addition, the optic nerve gets thinner and the cells of the lateral geniculate ganglion disappear partially. On average, ocular blood flow (OBF) is reduced in glaucoma patients in various tissues of the eye. Increased intraocular pressure (IOP) is a major risk factor for glaucomatous damage. Nevertheless, there is little doubt that other risk factors besides IOP are involved. One such risk factor is a primary vascular dysregulation (PVD) occurring in patients with a disturbed autoregulation, another risk factor is oxidative stress.

## Introduction

Gingko biloba has existed for over 250 million years and is indigenous to Korea, Japan, and China, but can be found worldwide. It may grow to 40 m, and live for over 1,000 years. The extracts of the gingko biloba leaves have been used for hundreds of years to treat various disorders such as asthma, vertigo, fatigue, and tinnitus or circulatory problems [1-3].

These extracts consist mainly of flavonoids and terpenoids. Two of the main extracts are EGb761 and LI 1370. Most pharmacological, toxicological, and clinical studies have focused on the neuroprotective value of these two main extracts [4-6].

Neuroprotection is a rapidly expanding area of research. This area is of particular interest due to the fact that it represents a new avenue of therapy for a frustrating disease that may progress despite optimal treatment [7]. One such disease is glaucoma.

Glaucoma leads to the loss of retinal ganglion cells and their axons but also to tissue remodeling which involves both the optic nerve head and the retina. In the retina, the astrocytes get activated. In addition, the optic nerve gets thinner and the cells of the lateral geniculate ganglion disappear partially [8]. On average, blood flow is reduced in glaucoma patients in various tissues of the eye. Blood flow reduction is more pronounced in normal tension glaucoma (NTG) than in high tension glaucoma (HTG) and comparatively, more in patients with progressive types of glaucoma than those with stable forms of glaucoma [9].

Increased intraocular pressure (IOP) is a major risk factor for glaucomatous damage and it is well established that an IOP reduction improves, on average, the prognosis of all types of glaucoma. Nevertheless, there is little doubt that other risk factors besides IOP are involved so that even an ideal IOP does not stop progression in all patients [10]. The objective of this review is to provide a scientific opinion on the indications for Ginkgo biloba as an adjuvant therapy for normal tension glaucoma patients and for high tension glaucoma patients progressing despite a normalized IOP.

## Pharmacological properties of Ginkgo biloba extract

### Antioxidative effects

Ginkgo contains many different flavonoids, including polyphenolic flavanoids which have been proven to exert antioxidative properties by delivering electrons to free radicals [11].

Many compounds, such as e.g., vitamins E and C also have antioxidative properties. The particularity of Ginkgo biloba extract is that unlike vitamins E and C, the polyphenolic flavanoids are able to act at the mitochondrial level.

**Figure 1:**
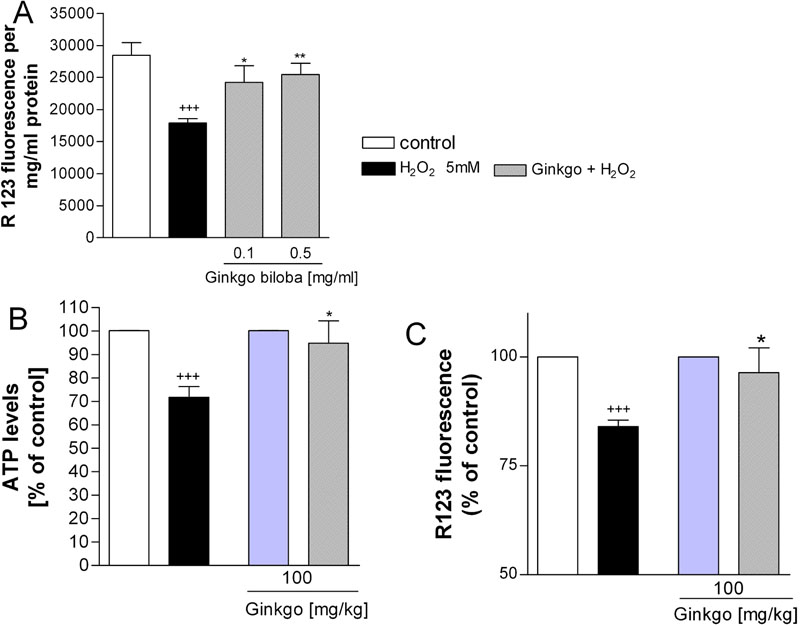
Stabilization of mitochondrial membrane potential. **A**: In vitro treatment with EGb 761 after H_2_O_2_ insult improves the reduction of mitochondrial membrane potential in dissociated mouse brain cells. Brain cells were damaged with H_2_O_2_ (5 mM) for 1 h; then EGb 761 was added for 6 h. **B**: In vivo treatment with EGb 761 improves the decrease of ATP levels in dissociated brain cells. Treated animals received 100 mg/kg EGb 761 p.o. once daily for 2 weeks. Control animals were treated with placebo (0.9% NaCl solution). ATP levels were measured after a 2 h incubation of dissociated mouse brain cells with 2mM H_2_O_2_. **C**: In vivo treatment with EGb761 improves mitochondrial membrane potential of isolated mitochondria after H_2_O_2_ insult. Treated animals received 100 mg/kg EGb 761 p.o. once daily for 2 weeks. Control animal were treated with placebo (0.9% NaCl solution). Mitochondrial membrane potential was measured after a 2 h incubation with 2 mM H_2_O_2_. Reproduced with permission from Eckert et al. [12].

In an in vitro study, PC12 cells were used to examine the protective features of EGb761 on mitochondria stressed with hydrogen peroxide and antimycin, an inhibitor of complex III [12] (Figure 1). In addition, the efficacy of EGb761 in the Abeta-induced 3-(4,5 dimethylthiazol-2-yl)-2,5-diphenyltetrazolium bromide (MTT) reduction in PC12 cells was examined. The authors of the study also examined the effects of EGb761 on reactive oxygen species (ROS) levels and ROS-induced apoptosis in lymphocytes from aged mice after in vivo administration. EGb761 was able to protect mitochondria from the attack of hydrogen peroxide, antimycin and Abeta. Furthermore, EGb761 reduced ROS levels and ROS-induced apoptosis in lymphocytes from aged mice treated orally with EGb761 for 2 weeks. These data support neuroprotective properties of EGb761, such as protection against Abeta-toxicity and antiapoptotic properties which are probably due to Ginkgo’s antioxidative effect at the mitochondrial level.

### Stabilization of the mitochondria

The mitochondria play a major role in several diseases, particularly neurodegenerative diseases, including glaucoma.

Indeed Abu-Amero et al. [13] revealed several mitochondrial abnormalities in patients with glaucoma. They examined 27 patients with definite chronic glaucoma patients and analyzed the following parameters: a) the myocilin (*MYOC*) and the optineurin (*OPTN*) genes were sequenced, b) the entire mitochondrial DNA (mtDNA) coding region was sequenced, c) relative mtDNA content was investigated, and d) the mitochondrial respiratory function was assessed. The authors of the study found only three benign polymorphisms, which were identified in *MYOC* and *OPTN* in patients with primary open-angle glaucoma (POAG) and in control subjects. Conversely, 27 different novel non-synonymous mtDNA changes were found, only in patients with POAG (not control subjects), 22 of which (found in 14 patients) were potentially pathogenic. mtDNA content was relatively increased in 17 patients with POAG compared with age-matched control subjects, also implying a possible response to oxidative stress. Mean mitochondrial respiratory activity was decreased significantly by 21% in patients with glaucoma compared with control subjects. These results reveal a spectrum of mitochondrial abnormalities in patients with glaucoma and may open up new experimental and therapeutic opportunities for glaucoma.

Besides, oxidative damage in mitochondria is particularly relevant for the following reasons: a) the mitochondria themselves are major sources of free radicals, b) mitochondria have a reduced DNA repair capacity, c) an oxidative damage leads to reduced membrane potential and thereby a reduced ATP-production; this shifts the cell in a low energy state and makes the cell more vulnerable to other damaging factors, d) if the damage of the mitochondria exceeds a certain level, cytochrome C is released and this molecule then starts a chain reaction leading to apoptosis.

Ginkgo has been proven to act at the mitochondrial level, by stabilizing the inner membrane and increasing the membrane potential, restoring the respiratory chain and increasing ATP-production. Abdel-Kader et al. [14] used PC12 cells, dissociated mice brain cells, as well as isolated mitochondria to investigate the effects of EGb761 on mitochondrial functions. They mimicked mitochondrial abnormalities during aging by using external factors (nitrosative stress, serum deprivation and complexes inhibitors) which are altering mitochondrial processes, such as energy metabolism. As markers for the function of mitochondria, ATP levels and mitochondrial membrane potential were measured. EGb761 alleviated mitochondrial functions in vitro at concentrations as low as 0.01 mg/ml. Treating two different age groups of mice with EGb761 (100 mg/kg bodyweight for 14 days) showed beneficial effects on complexes I, IV, and V of the mitochondrial respiratory chain and against nitrosative stress. Interestingly, these effects were only observed in the aged mice group, proving higher efficacy of Ginkgo during aging. The single components of Ginkgo extract showed a protective effect in both cell models of the mitochondrial membrane potential as well. This indicates that the components of Ginkgo act in a complementary manner to enable mitochondria stabilization.

### Anti-inflammatory effects

Many diseases, including neurodegenerative diseases, have a certain inflammatory component. These are normally not acute inflammations, but rather low-grade inflammations affecting certain cells, like for e.g., the glial cells in the brain. When these cells are activated, they produce inflammatory molecules such as TNFα, NOS-2, COX-2, etc.

Ginkgo extract has been shown to reduce the activation of cells (e.g., when activated through ischemia/reperfusion); under treatment with EGb761, NOS-2 is less upregulated and thereby less NO is produced. NO is a benign molecule, that has several important physiologic functions. However, if NO production is increased in areas with oxidative stress, NO^-^ fuses with O2^-^, thus releasing ONOO^-^, a highly cell damaging molecule [10].

In their study, Varga et al. [15] could demonstrate the dual effect of EGb761: it decreases the production of O2- and reduces the production of NO.

### Rheological effects

Rheological alterations occur in several clinical conditions. These alterations affect the macrocirculation to a lesser extent, while it has a significant effect on microcirculation. The relevance of the rheological alteration in ocular disease has been investigated by Sofi F et al. [16]. The authors of this article studied the hemorheological profile in 180 patients with retinal vein occlusion (RVO) and in 180 healthy subjects comparable for age and gender. Their data indicate that an alteration of hemorheological parameters may modulate the susceptibility to the RVO. Ginkgo promotes erythrocytes deformability, decreases fibrinogen levels, improves blood viscosity and viscoelasticity.

Indeed Huang et al. [17] demonstrated the improvement in abnormal hemorheological parameters after ingestion of Ginkgo biloba extract. Hemorheological parameters were measured before and 3 months after EGb761 oral ingestion in 25 type 2 diabetic patients with retinopathy. After taking EGb761 orally for 3 months, the blood viscosity was significantly reduced, viscoelasticity was significantly reduced in diabetic patients, the level of erythrocyte malondialdehyde (MDA) was reduced by 30%, and the deformability of erythrocyte was increased by 20%. Lastly, retinal capillary blood flow rate increased significantly. The authors of the study conclude that oral administration of EGb761 enables to improve hemorheological parameters, which in turn may facilitate blood perfusion.

### Antithrombotic properties

Ginkgo extract has an antithrombotic effect. However, the actual data do not corroborate the hypothesis Ginkgo extract may significantly increase the risk of clinically relevant and dangerous hemorrhages [18].

### Vasorelaxative properties

Ginkgo biloba increases microcirculation by improving the endothelium dependent vasodilation, as shown by the study conducted by Wu et al. [19].

In their study, the effect of Ginkgo on distal left anterior descending coronary artery (LAD) blood flow and endothelium-dependent brachial artery flow-mediated dilation (FMD) was tested in healthy elderly adults. Sixty healthy elderly adults were randomly assigned to either Ginkgo biloba extract (GBE) or control groups. LAD blood flow and brachial artery FMD were measured non-invasively using high-resolution ultrasound before and after intravenous administration of GBE or saline. GBE significantly increased LAD blood flow in maximal diastolic peak velocity (MDPV), maximal systolic peak velocity (MSPV) and diastolic time velocity integral (DTVI) compared with the placebo group. Brachial artery FMD was also significantly increased. A linear correlation was found between the percentage change in MDPV, MSPV, or DTVI of LAD blood flow and the percentage change in brachial artery FMD following treatment with GBE. These data demonstrate that GBE treatment in healthy elderly adults leads to the increase of LAD blood flow in MDPV, MSPV and DTVI, and the increased response might relate to the improved endothelium-dependent vasodilatory capacity.

### Antivasospastic properties

In many clinical conditions, particularly in glaucoma, vasospasms play a crucial role [20].

The antivasospastic property of Ginkgo biloba has clearly been demonstrated by Bayar et al. [21]. The authors of the study investigated the effects of EGb761 on basilar artery vasospasm in an experimental canine subarachnoid hemorrhage model. Morphometric analyses were performed, and serum and cerebrospinal fluid endothelin-l levels were measured by radioimmunoassay. Comparisons were made between treated and untreated groups. Twenty-four mongrel dogs were randomly assigned to three groups. The animals in group 1 (n=8) were not subjected to subarachnoid hemorrhage and received no treatment. In this group, serum and cerebrospinal fluid endothelin-l levels were measured daily for 8 days. On day 9, the animals were killed and their basilar arteries were excised for histopathological examination. In group 2 (n=8), subarachnoid hemorrhage was produced using autologous arterial blood, and daily intravenous boluses of saline were administered for the next 8 days. Assessments of endothelin-l levels and the basilar arteries were performed as described for group 1. In group 3 (n=8), subarachnoid hemorrhage was produced using autologous arterial blood, and daily intravenous boluses of EGb761 were administered for 8 days. Endothelin-1 levels and the basilar arteries were assessed as described above. The groups' serum endothelin-1, cerebrospinal fluid endothelin-1, and histopathological findings were compared. In group 3, the serum and cerebrospinal fluid endothelin-1 levels followed the same pattern observed in group 2; however, the arteries showed significantly less vasospasm than that observed in group 2. The results clearly show that intravenous boluses of EGb761 decreases morphologic vasospasm in the dog basilar artery. This finding is of great interest, as endothelin is increased in glaucoma patients, particularly in patients with normal tension glaucoma.

## Gingko biloba and glaucoma

### Pathophysiology of glaucoma

Ginkgo biloba positively influences oxidative stress and disturbed vascular circulation. Both reduced microcirculation and oxidative stress are involved in the pathogenesis of glaucoma. Therefore, already from a theoretical point of view, the pharmacological properties of Ginkgo can be expected to be beneficial for eye as well. Before focusing on the potential benefit of Ginkgo for glaucoma in the following sections, the pathomechanisms involved in the ocular disease induced by oxidative stress and/or disturbed microcirculation are summarized below.

When discussing glaucoma, there is a need to clearly separate mechanisms leading to an increase in intraocular pressure (IOP) and mechanisms that lead to glaucomatous damage also known as glaucomatous optic neuropathy (GON).

There are different aspects to the pathomechanisms in both conditions. Oxidative stress is involved in the pathogenesis of IOP, whereas for GON, factors associated with disturbed vascular regulation play a major role. Both aspects may potentially be influenced by a Ginkgo therapy and are discussed in more detail below.

#### Intraocular pressure (IOP)

Oxidative stress has been implicated to be a cause of increased intraocular pressure by triggering trabecular meshwork (TM) degeneration and thus contributing to alterations in the aqueous outflow pathway [22,23]. The human TM is composed of collagen lamellae lined by endothelial cells. The space between the collagen beams of the TM is filled with extracellular matrix, composed mostly of glycoproteins and proteoglycans, where the aqueous humor filters through [24,25]. The TM is in constant contact with the aqueous humor from which ROS may be generated through light catalyzed reactions, metabolic pathways or inflammation [22,24,26,27]. Disturbance of the TM cell status by an insult such as oxidative stress, may lead to cellular loss and an overexpression or alteration in the structures of various glycoproteins in the extracellular matrix [25,28,29], which interfere with the TM function, and lead to impaired aqueous humor outflow and thereby an increase in IOP.

The pathogenic role of oxidative stress in increasing IOP by reducing aqueous outflow facility, is supported by various experimental studies performed in vitro and in vivo. In vitro treatment of human TM cells with hydrogen peroxide alters cellular adhesion and integrity [25]. In an animal study of calf, perfusion of TM cells with peroxide has shown to reduce aqueous humor drainage from the anterior chamber of the calf's eye [30]. In humans, oxidative DNA damage has been reported to be significantly higher in the TM cells of glaucoma patients than in those of age-matched controls [31]. Further studies demonstrate abundant oxidative nucleotide modification (8-OH-dG) levels in human TM to be significantly correlated to the increase in IOP and to visual field damage [30,32]. Further evidence suggests that patients with POAG exert mitochondrial abnormalities implicating that mitochondrial dysfunction is most probably a consequence of oxidative stress [33]. Free radicals, contained in the aqueous humor contribute to pathogenic alterations in the TM [34]. Findings show resistance to the outflow of aqueous humor of calf, as a result of TM cytoskeletal rearrangements and cellular loss, in the presence of increased levels of hydrogen peroxide [30]. The damage done by prooxidants to the aqueous humor outflow system may explain why radiologists more often suffer from ocular hypertension [35]. At the molecular level, human TM endothelium has been reported to be an enriched site of both endothelin and nitric oxide (NO) synthesis. Nitric oxide can interact with oxygen or metals such as copper or iron to modulate outflow resistance of the TM [36].

Moreover, findings suggest activity of the antioxidant defense in the aqueous outflow system: glaucoma patients display a significant depletion of total antioxidant potential in their aqueous humor [37] a decrease in plasmatic glutathione levels [38], and an increase in serum antibodies against glutathione-S- transferase [39]. The expression of endothelial–leukocyte adhesion molecule (ELAM-1), which provides protection against oxidative stress, is increased in the TM of glaucoma patients [40]. Another notable antioxidant, namely glutathione, is also found in high concentrations in both aqueous humor and in the TM of mammals [30,41]. In addition, the heat shock protein, alpha-B crystalline, which protects from oxidative damage, is overly expressed in TM cells of both human and monkey eyes stressed by heat [42]. All these changes can be primary or secondary of nature.

#### Glaucomatous optic neuropathy (GON)

Beside increased IOP, vascular damage and hypoxia are often associated with glaucoma. Interestingly, however, arteriosclerosis and its risk factors are only weakly associated with GON. The factors related to disturbed autoregulation, in particular a systemic primary vascular dysregulation (PVD), play in GON a more important role. These factors are best observed in normal tension glaucoma patients, as described by Kaiser et al. [43]. An insufficient autoregulation increases the risk for an unstable ocular perfusion, leading to an unstable oxygen supply. This unstable oxygen supply generates oxidative stress. As a consequence of oxidative stress, the concentration of superoxide (O2^-^) within the axons of the optic nerve head increases. The activation of the neighboring astrocytes by mechanical or by ischemic stress induces the production of nitric oxide (NO) in excess. NO diffuses into the axons and fuses with superoxide. The resulting peroxynitrate (ONOO^-^) diffuses within the axons toward the retina and the lateral geniculate nucleus and induces apoptosis [44].

The activation of the astrocytes has been shown to be another sign of oxidative stress in the optic nerve of glaucoma patients [45].

These data indicates that beside IOP, disturbed regulation of microcirculation and oxidative stress are major risk factors leading to GON. Both aspects can be influenced by Ginkgo.

### Pre-clinical data for glaucoma

#### Effect of Ginkgo biloba on glaucomatous optic neuropathy

Normal-tension glaucoma, is characterized by progressive optic nerve damage and visual field loss with a statistically normal intraocular pressure. Normal-tension glaucoma is thought to be related, at least in part, to dysregulated blood flow of the optic nerve [46].

There are animal models for high tension glaucoma but neither for normal tension glaucoma nor for high tension glaucoma that progresses despite normalized IOP.

Although robust animal models are lacking, Ginkgo is used by glaucoma specialists for treatment of patients with normal tension glaucoma and for patients with a glaucoma that progresses despite IOP lowering treatment. This use is motivated by the fact, that on the one hand, there are currently no other therapeutic options for these cases and on the other hand, the pharmacological profile of Ginkgo exactly fits to the pathophysiology of these special conditions.

In this context, it is however necessary to emphasize that a classical separation between pre-clinical and clinical data are difficult or impossible. While classical animal models with increased IOP do exist [47,48], there are no animal models available for the following two conditions: a) for normal tension glaucoma and b) for glaucoma that progresses despite a normalized IOP. However, these conditions are exactly the ones in which microcirculation and oxidative stress are of particular relevance and therefore potentially treatable with Ginkgo.

Pre-clinical data in a traditional sense are not directly available to assess the beneficial effect of Ginkgo for a) normal tension glaucoma and b) glaucoma that progresses despite a normalized IOP. It is, however, possible to make meaningful extrapolations from other pathological conditions (although they may not always be related with the ocular organ).

As summarized by Tezel G, oxidative stress is clearly is involved in the pathogenesis of glaucoma [49].

Reactive oxygen species (ROS) are generated as by-products of cellular metabolism, primarily in the mitochondria. Although ROS are essential participants in cell signaling and regulation, when their cellular production overwhelms the intrinsic antioxidant capacity, damage to cellular macromolecules such as DNA, proteins, and lipids ensues. Such state of “oxidative stress” is thought to contribute to the pathogenesis of several neurodegenerative diseases. Growing evidence supports the involvement of oxidative stress as a common component of glaucomatous neurodegeneration in different subcellular compartments of retinal ganglion cells (RGCs) [50-52]. Besides the evidence of direct cytotoxic consequences leading to RGC death, it also seems highly possible that ROS are involved in signaling RGC death. In this signaling pathway, ROS act as a second messenger and/or modulates protein function by redox modifications of downstream effectors through enzymatic oxidation of specific amino acid residues [53]. Different studies provide cumulating evidence, which supports the association of ROS with different aspects of the neurodegenerative process [54-56]. Oxidative protein modifications during glaucomatous neurodegeneration increase neuronal susceptibility to damage and also lead to glial dysfunction [57]. Oxidative stress-induced dysfunction of glial cells may contribute to spreading neuronal damage by secondary degeneration [58]. Oxidative stress also promotes the accumulation of advanced glycation and products in glaucomatous tissues [59]. In addition, oxidative stress takes part in the activation of immune response during glaucomatous neurodegeneration [60], as ROS stimulate the antigen presenting ability of glial cells and also function as co-stimulatory molecules during antigen presentation.

On one hand, there is clear evidence that oxidative stress plays a role in the pathogenesis of GON and on the other hand, there is enough evidence with regard to the antioxidative property of Ginkgo biloba.

The potential value of antioxidative treatment in glaucoma was recently summarized by Mozaffarieh et al. [61]. The authors consider antioxidative treatment meaningful in glaucoma even though the clinical evidence is sparse.

Several peer-reviewed data support the involvement of mitochondria dysfunction in the pathogenesis of glaucoma [60,62,63].

Evidence regarding the improvement of mitochondrial function with Ginkgo exists. EGb761 given shortly after initiating mitochondrial damage by sodium nitroprusside (nitric oxide donor) improved the mitochondrial membrane potential of PC12 cells significantly and dose dependently. Under these conditions, EGb761 also reversed the decrease in ATP production. These findings clearly show stabilization and protection of mitochondrial function as a specific and very sensitive property of EGb761 at therapeutically relevant doses [12]. In an in vitro study EGb761 alleviated mitochondrial functions (ATP levels and mitochondrial membrane potential) at concentrations as low as 0.01 mg/ml 14].

Disturbed microcirculation plays a crucial role in the pathogenesis of glaucomatous damage [10]. An unstable ocular perfusion, either due to IOP fluctuation or a disturbed autoregulation (due to primary vascular dysregulation syndrome), leads to a mild reperfusion injury. The fusion of superoxide (O_2_^.-^) anion (produced in the mitochondria of the axons) with the nitric oxide (NO; diffusing from the astrocytes) results in the production of peroxynitrite (ONOO^–^). It is possible that the diffusion of endothelin and metalloproteinases to the surrounding of the optic nerve head leads to a local vasoconstriction. This vasoconstriction increases the risk for venous occlusion and weakens the blood-brain barrier, which in extreme situations results in splinter hemorrhages. The involvement of primary vascular dysregulation in the pathogenesis of glaucomatous optic neuropathy may explain why women, as well as Japanese, suffer more often from normal-tension glaucoma.

It is known that ginkgo improves microcirculation in the brain and it may therefore be assumed that ginkgo exerts a similar effect in the eye. Zhang et al. [64] investigated the therapeutic effect of EGb761 on hypertension and its possible mechanisms in the view of cerebral microcirculation in twenty normotensive rats and 24 spontaneously hypertensive rat (SHR) rats. They suggested that EGb761 had therapeutic effect on SHR rats by increasing blood perfusion, regulating vasomotion function, opening efficiently capillaries and releasing the peripheral resistance. It was concluded that EGb761 could be used to regulate hypertension and to protect the cerebral microcirculatory function.

Although the investigation of Zhang et al. was done at the cerebral level, it may be assumed that similar conclusion may be drawn for the eye.

A mild but repeated reperfusion injury (either due to IOP fluctuating on a level that momentarily exceeds autoregulation capacity or due to disturbed autoregulation) leads to the oxidative stress and thereby to glaucomatous damage on the long run [65].

Ginkgo reduces ischemia/reperfusion injury in the ischemic/reperfused diabetic rat retina. This has been demonstrated by the free radical scavenging property of Ginkgo [66]. Hao et al. [67] studied the role of Ginkgo in the hearts of rats with from ischemia-reperfusion. They investigated the function of isolated hearts subjected to ischemia-reperfusion (IR) with or without Ginkgolide B (GB) pretreatment. They found that GB exposure improved the function of left ventricle from IR injury and decreased infarct size and the release of lactate dehydrogenase (LDH). The results of the study showed that GB could partly prevent IR injury in rat heart.

Based on the results observed in rat diabetic retina and ischemia-reperfusion in rat heart, it can be assumed that a similar beneficial effect of ginkgo occurs in the optic nerve head of glaucoma patients, as these patients also suffer from ischemia-reperfusion injury.

Optic disc hemorrhages are associated with a higher risk for progression. Normal tension glaucoma is particularly affected by these hemorrhages [68].

However the frequency of these hemorrhages cannot be reduced by IOP lowering treatment [69] and there is no treatment available at present to reduce this risk factor. It is known, however, that Ginkgo (EGb761), can prevent vasospasm in subarachnoidal hemorrhages [70]. Therefore it can be assumed that Ginkgo has also a beneficial effect in glaucoma patients with optic disc hemorrhages.

As mentioned above, animal models for normal tension glaucoma – or for glaucoma progressing despite a normalized IOP – do not exist. Nevertheless, even in classical high tension glaucoma animal models, ginkgo has been shown to reduce glaucomatous damage [71].

#### High tension glaucoma

**Figure 2:**
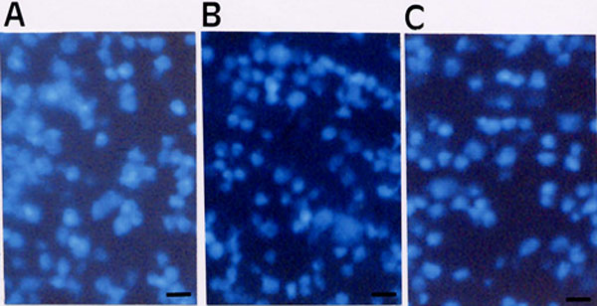
Surviving retinal ganglion cells distribution in the retina in a rat model of chronic glaucoma. Three groups of animals: **A**: control, **B**: EGb761-treated for 5 months, and **C**: vehicle-treated. A difference between surviving retinal ganglion cells distribution in eyes with elevated IOP that were treated with EGb 761 and vehicle. Reproduced with permission from Hirooka et al. [71].

**Figure 3:**
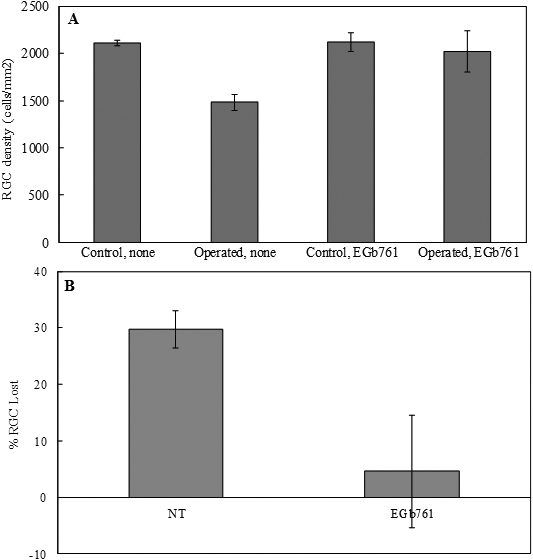
The effect of gingko extract (EGb76) on retinal ganglion cells of rats. **A**: Retinal ganglion cells density in retinas with chronic, moderately elevated IOP. Retinal ganglion cells (RGC) were counted in the peripheral retina, approximately 4.0 mm from the optic disc. The graph depicts the mean±standard deviation of five animals treated with vehicle. A significant difference between retinal ganglion cell densities in eyes with elevated IOP that were treated with EGb 761 and vehicle was evident (p=0.0007). Four groups of animals: 1) Control without treated, 2) eyes with elevated IOP without treated (Operated, none), 3) Control, treated with EGb 761, 4) eyes with elevated IOP, treated ith EGb 761 (Operated, EGb761). **B**: Retinal ganglion cells lost at 5 months in rat eyes with chronic, moderately elevated IOP. Two groups: 1) Not treated, 2) Treated with EGB761. Reproduced with permission from Hirooka et al. [71].

Unlike for normal tension glaucoma, an accepted therapy for high tension glaucoma exists, namely the IOP lowering treatment. In addition, in the case of high tension glaucoma, factors like disturbed microcirculation or oxidative stress seem to play a less important role than in normal tension glaucoma. The fact that ginkgo had a clearly beneficial effect even in animals with high tension glaucoma is therefore even more remarkable: the effect of Ginkgo biloba extract against neurotoxicity of retinal ganglion cells was investigated in rats with chronic moderately elevated intraocular pressure (IOP) [71]. Unilateral chronic moderately elevated IOP was produced in rats by cautery of three episcleral vessels. Secondary degeneration was measured with and without EGb761 treatment for 5 months. After 5 months, the retinal ganglion cells loss between the EGb761 treated group and the control group (no EGb761 treatment) was compared. The authors of the study concluded that pre-treatment and early post-treatment with EGb761 is an effective neuroprotectant in a rat model of chronic glaucoma (Figure 2 and Figure 3).

#### Overall conclusion from the experimental data for Ginkgo biloba and glaucoma

Based on the present knowledge of the pathogenesis of glaucomatous damage, Ginkgo interferes positively on different steps involved in the pathogenesis of glaucomatous damage (oxidative stress, microcirculation, mitochondrial function etc). However experimental models for normal tension glaucoma or for glaucomatous progression despite normal IOP do not exist. Therefore, the conclusions are based on extrapolation of other studies. And these extrapolations reveal a clear positive effect of Ginkgo.

But even in high tension glaucoma, where IOP plays a major role (whereas factors such as impaired microcirculation and oxidative stress are less relevant) and where an accepted therapy (namely the IOP lowering therapy) is available, the effect of ginkgo was highly significantly positive.

While all publications are indirectly in favor for Ginkgo, no single publication contradicts the positive effect of Ginkgo.

### Clinical data

Glaucoma is a chronic progressive disease, in which the damage develops over decades. Double blind controlled long-term studies are needed to definitively evaluate the value of a glaucomatous drug. However such controlled studies are money and time consuming. Therefore, very few controlled studies have been performed in glaucoma for any type of glaucoma treatment. To simplify the evaluation of the efficacy of so called glaucoma drugs, IOP is normally used as a surrogate in clinical trials. It is known from large scale studies, that IOP lowering treatment is particularly helpful for high tension glaucoma. To some extent, IOP lowering treatment is also helpful for normal tension glaucoma. However these studies have also demonstrated that a large part of patients (particularly in the group of normal tension glaucoma patients) progress despite a normalized IOP [46]. The progression of glaucoma often occurs even if a very low IOP has been reached by IOP lowering treatment. Therefore, there is an urgent need for an additional – non IOP lowering – glaucoma treatment.

The only such treatment that gained more or less general acceptance – even without definitive scientific proof – is a treatment with Ginkgo [72,73]. Glaucomatologists have been using Ginkgo for their patients successfully since many years.

From their point of view, Ginkgo is indicated a) in patients with normal tension glaucoma and b) in glaucoma patients that progress despite a normalized IOP. This indication is based on the fact that a) beside IOP, the pathogenic factors involved in the disease are disturbed microcirculation and oxidative stress, both conditions which can be influenced by Ginkgo. b) there are currently no alternative treatment for such cases. c) a significant positive effect in favor of Ginkgo was observed both in experimental glaucoma animals and in glaucoma patients.

#### Pathomechanisms involved in glaucoma

In glaucoma patients, ocular blood flow is reduced both in patients with normal tension glaucoma and in patients that progress despite a normalized IOP. Kaiser et al. [43] measured hemodynamic parameters in the ophthalmic artery, central retinal artery, central retinal vein, and lateral and medial short posterior ciliary arteries by color Doppler imaging in 237 patients with primary open-angle glaucoma and 124 age-matched normal control subjects. All patients showed a significant decrease in end-diastolic velocities and a significant increase in resistivity index in all arteries measured. Their data showed that hemodynamic parameters in the extraocular vessels are altered in patients with glaucoma.

Several studies have demonstrated that blood flow reduction has a negative predicting power for progression [74,75]. In a retrospective observational case studies, Satilmis et al. [76] evaluated the correlation between progression rate of glaucomatous damage and retrobulbar blood flow in an institutional setting. Twenty open-angle glaucoma patients with at least five visual field examinations and progressive damage in at least one eye were included in the study. As an indicator of progression rate of visual field damage, the angle to a horizontal line of the slope of the regression line of the visual field index mean defect over time was calculated for one randomly selected eye per patient. The association between this angle and intraocular pressure, as well as retro bulbar color Doppler imaging measurements, were analyzed by a multiple linear regression analysis in a stepwise forward approach. A faster rate in progression of glaucomatous damage was observed by a lower baseline end diastolic blood flow velocity in the central retinal artery and a higher baseline intraocular pressure. The rate of progression was not related to the extent of preexisting visual field damage and IOP. The authors of the study concluded that a significant correlation exists between the progression rate of glaucomatous visual field damage and retrobulbar hemodynamic variables.

#### Effects of Ginkgo on ocular microcirculation

Ginkgo improves ocular blood flow. Chung et al. [77] evaluated a possible therapeutic effect of Ginkgo biloba extract (GBE) on glaucoma patients who may benefit from improvements in ocular blood flow. A Phase I placebo-controlled crossover trial in 11 healthy volunteers was performed. Patients were treated with either GBE (40 mg) or placebo three times daily orally, for 2 days. Color Doppler imaging was used to measure ocular blood flow before and after treatment. There was a two week washout period between GBE and placebo treatment. Ginkgo biloba extract significantly increased end diastolic velocity (EDV) in the ophthalmic artery (OA), with no change seen in placebo. No side effects related to GBE were found. GBE did not alter arterial blood pressure, heart rate, or IOP. The authors of the study concluded that GBE significantly increased EDV in the OA. According to them, the beneficial properties of GBE on ocular blood flow deserve further investigation as a potential treatment of glaucomatous optic neuropathy (as well as other ischemic ocular diseases).

Although the experimental data have been performed with healthy subjects, these data support the finding that Ginkgo may be beneficial for glaucomatous disease where ocular microcirculation is impaired.

#### Novel strategies for Glaucoma treatment

While classic glaucoma treatment focuses on IOP reduction, open question remains, as cases exist for which a reduction of IOP does not stop the progression of the disease. Besides, a better knowledge of the pathogenesis of the disease has opened up new therapeutical approaches. The different therapeutic options, which are targeted toward factors other than. The non-IOP lowering drugs described in the review are targeted at different levels of the pathophysiology involved in glaucomatous optic neuropathy, such as: inhibition of the activation of astrocytes, inhibition of nitric-oxide synthase 2, improvement of vascular regulation, counteract oxidative stress, inhibition of matrix metalloproteinase, upregulation of heat shock protein, neuroprotection [78].

Positive preclinical data have been obtained with drugs inhibiting the activation of astrocytes, nitric-oxide synthase 2 inhibitor, matrix metalloproteinase-9 inhibitor, drugs with neuroprotective properties and with the upregulation of heat shock proteins. However, data supporting their potential effectiveness in glaucoma treatment in human are not yet available.

In small open cohort studies, the improvement of ocular blood flow with carbonic anhydrase inhibitors, like acetazolamide, has shown to improve visual fields in glaucoma patients [79,80]. Similar results were also observed with calcium channel blockers in patients with vascular dysregulation [81]. Besides, in a placebo-controlled, double-blind study, treatment with Ginkgo biloba has induced some reversibility of visual field damage in patient with normal tension glaucoma.

As the field of vision is affected at the early stages of glaucomatous disease, the visual fields are the most important parameter to quantify glaucomatous damage.

In a placebo controlled double blind study, treatment with ginkgo has also induced some reversibility of visual field damage in normal tension glaucoma [82].

**Figure 4:**
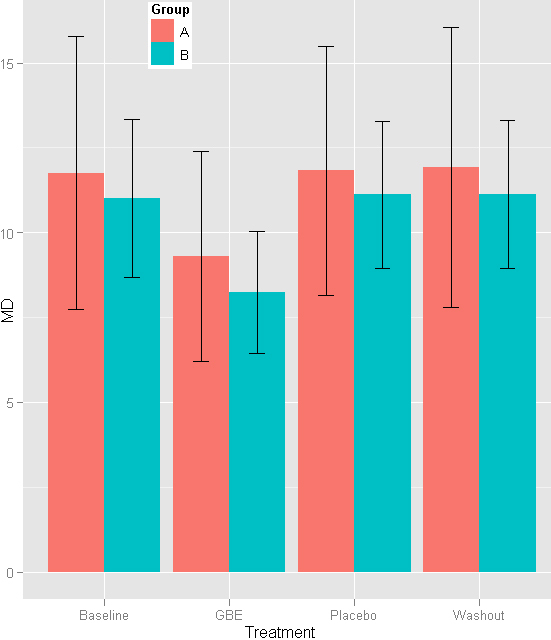
Improvement in preexisting visual field damage in patients with normal tension glaucoma after treatment with GBE. Patients are divided in two groups according to sequences of treatment. Group A: sequence of treatment: GBE- wash out- placebo. Group B: sequence of treatment: placebo-washout-GBE. GBE=Gingko biloba. Reproduced with permission from Quaranta et al. [82].

The authors of the study evaluated the effect of Ginkgo biloba extract (GBE) on preexisting visual field damage in patients with normal tension glaucoma (NTG) with the help of a prospective, randomized, placebo-controlled, double-masked crossover trial. Twenty-seven patients with bilateral visual field damage resulting from NTG participated. Patients received 40 mg GBE, administered orally, three times daily for 4 weeks, followed by a wash-out period of 8 weeks, then 4 weeks of placebo treatment. Other patients underwent the same regimen, but took the placebo first and the GBE last. Visual field tests, performed at baseline and at the end of each phase of the study, were evaluated for changes. Changes in visual field and any ocular or systemic complications were the main outcome measures. After GBE treatment, a significant improvement in visual fields indices was recorded. Neither ocular nor systemic side effects were observed for the duration of the trial. It can be concluded from this study that the administration of Ginkgo biloba extract improves preexisting visual field damage in some patients with NTG (Figure 4).

Beside increased IOP, disturbed microcirculation and oxidative stress are involved in the pathogenesis of glaucoma. Ginkgo has clearly been proven to improve microcirculation and the antioxidative capacity. Moreover, the main damage in glaucoma occurs in the mitochondria and Ginkgo clearly has a stabilizing effect on the mitochondria. Optic disc hemorrhages often occur in glaucoma patients and are associated with reduced prognosis. By extrapolating the data obtained in the brain, it can be assumed that ginkgo has also a beneficial effect in optic disc hemorrhages. Preclinical data have shown the protective effect of ginkgo on the retinal ganglion cells in experimental glaucomatous animals. Moreover, the potential beneficial value of Ginkgo has been described in one clinical double-blind study, in which Ginkgo has even induced some reversibility of glaucomatous visual field damage. With the exception of a single study which investigated the hemodynamic parameters after a single administration of Ginkgo biloba, no other study revealed negative results.

## Safety

### Adverse reactions under Ginkgo biloba treatment

According to published studies and reports, the use of Ginkgo seems safe and well tolerated. Birks and Grimley cited in their meta-analysis that there were no differences between Ginkgo biloba and placebo in the proportion of participants experiencing any adverse event. The studies selected for this meta-analysis were administrating Ginkgo extract treatment up to six months. These data have been confirmed when standardized Ginkgo biloba was used in standard doses in clinical trials lasting from one up to six years [83-85].

In rare cases, mild gastrointestinal complaints, headache, and allergic skin reactions have been reported [86]. There are several published case reports linking Ginkgo to episodes of minor to severe bleeding. However, not all case reports clearly establish Ginkgo as the cause of bleeding. In most cases, other bleeding risk factors such as intake of other medications, old age, liver cirrhosis or recent surgery were also present. Bleeding occurred after several weeks or months of taking Ginkgo biloba [87].

To determine the effect of Ginkgo extract on coagulation parameters, Kohler et al. [88] assessed in a crossover study with fifty healthy volunteers the effect on bleeding time, coagulation parameters, or platelet activation and found no effect. Another placebo-controlled study evaluated the effects of Ginkgo biloba on homeostasis, coagulation and fibrinolysis when Gingko biloba extract were administered for two weeks. This study did not reveal any alteration of platelet function or coagulation [89]. Besides, large-scale clinical trials evaluating standardized Ginkgo leaf extracts on elderly patients showed that the incidence of bleeding in patients taking Ginkgo is not significantly higher than in those taking placebo [90,91].

### Interaction potential during Ginkgo biloba treatment

The risk of spontaneous bleeding may be increased when Ginkgo biloba extract is combined with nonsteroidal antiinflammatory drugs (NSAIDs) and anticoagulants such as heparin or warfarin [92]. In a recent randomized double-blind, placebo-controlled clinical trial, the effect of a co-medication of acetylsalicylic acid (ASA) and Ginkgo extract on platelet aggregation, bleeding time and coagulation parameters has been investigated [93]. ASA and the combination of ASA and Ginkgo biloba exerted similar effects on all coagulation parameters measured, including bleeding time and PAF induced platelet aggregation. Both treatments were well tolerated, and both number and nature of adverse events in the two groups were similar. These findings suggest that co-administration of ASA and Ginkgo biloba does not constitute a safety risk. According to the authors, this finding also applies for an elderly patient population undergoing treatment with Ginkgo biloba.

The potential interaction of a standardized Ginkgo extract with warfarin was tested in a placebo-controlled, crossover study involving 24 patients under a long-term warfarin treatment [94]. After administration of Ginkgo biloba, the mean dose of warfarin to maintain the target range for INR of 2.0–4.0 did not have to be changed during either treatment period. A population pharmacokinetic-pharmacodynamic modeling approach resulted in similar findings with regard to the potential of interaction during a co-medication with Ginkgo and warfarin [95].

In summary, although there were reports about individual cases of bleeding (whose direct relationship with Ginkgo biloba could not be established and which occurred mainly with high-risk patients), Ginkgo biloba appears to be predominantly safe in the long term use with no excess side effects compared with placebo.

## Conclusion

The classical treatment of glaucoma is and remains IOP reduction. However in normal tension glaucoma, the glaucomatous disease progresses despite a normal or normalized IOP. As the pharmacological properties of Ginkgo specifically target the factors involved in glaucomatous disease (disturbed ocular microcirculation, oxidative stress, impairment of mitochondrial function in the retinal ganglion cells), it can be assumed that Ginkgo could theoretically be beneficial for glaucoma.

Some authors did not find any association between Ginkgo biloba extract use and glaucoma or mentioned that little is known on the long-term impact of medication having an effect on ocular blood flow. However the date based on a) the pharmacological properties of ginkgo, b) in vitro-studies, c) animal studies and d) clinical studies provide cumulating evidence of the beneficial effect of Ginkgo for the above mentioned condition.

Ginkgo would probably be beneficial for all glaucoma patients. However the use of ginkgo can be recommended as an adjuvant therapy only for normal tension glaucoma patients and for high tension glaucoma patients progressing despite a normalized IOP. Such a limitation is economically meaningful and medically possible, as for these two conditions, there are no other therapeutic alternative so far (beside the IOP- lowering treatment).

Therefore, based on the current data set and the favorable safety profile of standardized Ginkgo biloba extract, the administration of a Ginkgo treatment could be considered in cases where glaucoma progresses despite a normal or normalized IOP.

## References

[1] Bachinskaya N, Hoerr R, Ihl R (2011). Alleviating neuropsychiatric symptoms in dementia: the effects of Ginkgo biloba extract EGb 761. Findings from a randomized controlled trial.. Neuropsychiatr Dis Treat.

[2] Li S, Tang D, Xue Z, Zhang Z, Sun X, Liu Y, Dong H, Yin X, Zhang Z (2011). Biphasic effect of EGb761 on simulated ischemia-induced rat BMSC survival in vitro and in vivo.. Life Sci.

[3] Janssen IM, Sturtz S, Skipka G, Zentner A, Garrido MV, Busse R (2010). Ginkgo biloba in Alzheimer's disease: a systematic review.. Wien Med Wochenschr.

[4] de Lima KC, Schilichting CL, Junior LA, da Silva FM, Benetoli A, Milani H (2006). The Ginkgo biloba extract, EGb 761, fails to reduce brain infarct size in rats after transient, middle cerebral artery occlusion in conditions of unprevented, ischemia-induced fever.. Phytother Res.

[5] Shah ZA, Nada SE, Dore S (2011). Heme oxygenase 1, beneficial role in permanent ischemic stroke and in Gingko biloba (EGb 761) neuroprotection.. Neuroscience.

[6] Chung SY, Cheng FC, Lee MS, Lin JY, Lin MC, Wang MF (2006). Ginkgo biloba leaf extract (EGb761) combined with neuroprotective agents reduces the infarct volumes of gerbil ischemic brain.. Am J Chin Med.

[7] Ritch R (2000). Neuroprotection: is it already applicable to glaucoma therapy?. Curr Opin Ophthalmol.

[8] Flammer J. Glaucoma a guide for patients an introduction for care-providers a reference for quick information. Bern: Hans Huber; 2001.

[9] Flammer J, Orgul S, Costa VP, Orzalesi N, Krieglstein GK, Serra LM, Renard JP, Stefansson E (2002). The impact of ocular blood flow in glaucoma.. Prog Retin Eye Res.

[10] Flammer J, Mozaffarieh M (2007). What is the present pathogenetic concept of glaucomatous optic neuropathy?. Surv Ophthalmol.

[11] Ou HC, Lee WJ, Lee IT, Chiu TH, Tsai KL, Lin CY, Sheu WH (2009). Ginkgo biloba extract attenuates oxLDL-induced oxidative functional damages in endothelial cells.. J Appl Physiol.

[12] Eckert A, Keil U, Scherping I, Hauptmann S, Muller WE (2005). Stabilization of Mitochondrial Membrane Potential and Improvement of Neuronal Energy Metabolism by Ginkgo Biloba Extract EGb 761.. Ann N Y Acad Sci.

[13] Abu-Amero KK, Morales J, Bosley TM (2006). Mitochondrial abnormalities in patients with primary open-angle glaucoma.. Invest Ophthalmol Vis Sci.

[14] Abdel-Kader R, Hauptmann S, Keil U, Scherping I, Leuner K, Eckert A, Muller WE (2007). Stabilization of mitochondrial function by Ginkgo biloba extract (EGb 761).. Pharmacol Res.

[15] Varga E, Bodi A, Ferdinandy P, Droy-Lefaix MT, Blasig IE, Tosaki A (1999). The protective effect of EGb 761 in isolated ischemic/reperfused rat hearts: a link between cardiac function and nitric oxide production.. J Cardiovasc Pharmacol.

[16] Sofi F, Mannini L, Marcucci R, Bolli P, Sodi A, Giambene B, Menchini U, Gensini GF, Abbate R, Prisco D (2007). Role of haemorheological factors in patients with retinal vein occlusion.. Thromb Haemost.

[17] Huang SY, Jeng C, Kao SC, Yu JJ, Liu DZ (2004). Improved haemorrheological properties by Ginkgo biloba extract (Egb 761) in type 2 diabetes mellitus complicated with retinopathy.. Clin Nutr.

[18] Sasaki Y, Noguchi T, Yamamoto E, Giddings JC, Ikeda K, Yamori Y, Yamamoto J (2002). Effects of Ginkgo biloba extract (EGb 761) on cerebral thrombosis and blood pressure in stroke-prone spontaneously hypertensive rats.. Clin Exp Pharmacol Physiol.

[19] Wu Y, Li S, Cui W, Zu X, Du J, Wang F (2008). Ginkgo biloba extract improves coronary blood flow in healthy elderly adults: role of endothelium-dependent vasodilation.. Phytomedicine.

[20] Flammer J, Pache M, Resink T (2001). Vasospasm, its role in the pathogenesis of diseases with particular reference to the eye.. Prog Retin Eye Res.

[21] Bayar MA, Erdem Y, Ozturk K, Bescalti O, Caydere M, Yucel D, Buharali Z, Ustun H (2003). The effect of EGb-761 on morphologic vasospasm in canine basilar artery after subarachnoid hemorrhage.. J Cardiovasc Pharmacol.

[22] Russell P, Johnson DH (1996). Enzymes protective of oxidative damage present in all decades of life in the trabecular meshwork, as detected by two-dimensional gel electrophoresis protein maps.. J Glaucoma.

[23] Saccà SC, Izzotti A, Rossi P, Traverso C (2007). Glaucomatous outflow pathway and oxidative stress.. Exp Eye Res.

[24] Ueda J, Wentz-Hunter K, Yue BY (2002). Distribution of myocilin and extracellular matrix components in the juxtacanalicular tissue of human eyes.. Invest Ophthalmol Vis Sci.

[25] Zhou L, Li Y, Yue BY (1999). Oxidative stress affects cytoskeletal structure and cell-matrix interactions in cells from an ocular tissue: the trabecular meshwork.. J Cell Physiol.

[26] Spector A, Garner WH (1981). Hydrogen peroxide and human cataract.. Exp Eye Res.

[27] Rose RC, Richer SP, Bode AM (1998). Ocular oxidants and antioxidant protection.. Proc Soc Exp Biol Med.

[28] Li AF, Tane N, Roy S (2004). Fibronectin overexpression inhibits trabecular meshwork cell monolayer permeability.. Mol Vis.

[29] Wentz-Hunter K, Shen X, Okazaki K, Tanihara H, Yue BY (2004). Overexpression of myocilin in cultured human trabecular meshwork cells.. Exp Cell Res.

[30] Kahn MG, Giblin FJ, Epstein DL (1983). Glutathione in calf trabecular meshwork and its relation to aqueous humor outflow facility.. Invest Ophthalmol Vis Sci.

[31] Izzotti A, Sacca SC, Cartiglia C, De FS (2003). Oxidative deoxyribonucleic acid damage in the eyes of glaucoma patients.. Am J Med.

[32] Saccà SC, Pascotto A, Camicione P, Capris P, Izzotti A (2005). Oxidative DNA damage in the human trabecular meshwork: clinical correlation in patients with primary open-angle glaucoma.. Arch Ophthalmol.

[33] Abu-Amero KK, Morales J, Bosley TM (2006). Mitochondrial abnormalities in patients with primary open-angle glaucoma.. Invest Ophthalmol Vis Sci.

[34] Alvarado J, Murphy C, Juster R (1984). Trabecular meshwork cellularity in primary open-angle glaucoma and nonglaucomatous normals.. Ophthalmology.

[35] Scurti D, L'Abbate N, Capozzi D, Lofrumento R, Crivellini S, Ambrosi L (1992). Ocular hypertension in radiologists and radiology technicians.. Med Lav.

[36] Haefliger IO, Dettmann E, Liu R, Meyer P, Prunte C, Messerli J, Flammer J (1999). Potential role of nitric oxide and endothelin in the pathogenesis of glaucoma.. Surv Ophthalmol.

[37] Ferreira SM, Lerner SF, Brunzini R, Evelson PA, Llesuy SF (2004). Oxidative stress markers in aqueous humor of glaucoma patients.. Am J Ophthalmol.

[38] Gherghel D, Griffiths HR, Hilton EJ, Cunliffe IA, Hosking SL (2005). Systemic reduction in glutathione levels occurs in patients with primary open-angle glaucoma.. Invest Ophthalmol Vis Sci.

[39] Yang J, Tezel G, Patil RV, Romano C, Wax MB (2001). Serum autoantibody against glutathione S-transferase in patients with glaucoma.. Invest Ophthalmol Vis Sci.

[40] Wang N, Chintala SK, Fini ME, Schuman JS (2001). Activation of a tissue-specific stress response in the aqueous outflow pathway of the eye defines the glaucoma disease phenotype.. Nat Med.

[41] Richer SP, Rose RC (1998). Water soluble antioxidants in mammalian aqueous humor: interaction with UV B and hydrogen peroxide.. Vision Res.

[42] Tamm ER, Russell P, Johnson DH, Piatigorsky J (1996). Human and monkey trabecular meshwork accumulate alpha B-crystallin in response to heat shock and oxidative stress.. Invest Ophthalmol Vis Sci.

[43] Kaiser HJ, Schoetzau A, Stumpfig D, Flammer J (1997). Blood-flow velocities of the extraocular vessels in patients with high-tension and normal-tension primary open-angle glaucoma.. Am J Ophthalmol.

[44] Mozaffarieh M, Grieshaber MC, Flammer J (2008). Oxygen and blood flow: players in the pathogenesis of glaucoma.. Mol Vis.

[45] Hernandez MR, Miao H, Lukas T (2008). Astrocytes in glaucomatous optic neuropathy.. Prog Brain Res.

[46] Flammer J (1990). Normal-pressure glaucoma.. Fortschr Ophthalmol.

[47] Quigley HA, Cone FE, Gelman SE, Yang Z, Son JL, Oglesby EN, Pease ME, Zack DJ (2011). Lack of neuroprotection against experimental glaucoma in c-Jun N-terminal kinase 3 knockout mice.. Exp Eye Res.

[48] Millar JC, Clark AF, Pang IH (2011). Assessment of aqueous humor dynamics in the mouse by a novel method of constant-flow infusion.. Invest Ophthalmol Vis Sci.

[49] Tezel G (2006). Oxidative stress in glaucomatous neurodegeneration: mechanisms and consequences.. Prog Retin Eye Res.

[50] Ganapathy PS, White RE, Ha Y, Bozard BR, McNeil PL, Caldwell RW, Kumar S, Black SM, Smith SB (2011). The role of N-methyl-D-aspartate receptor activation in homocysteine-induced death of retinal ganglion cells.. Invest Ophthalmol Vis Sci.

[51] Li GY, Fan B, Su GF (2009). Acute energy reduction induces caspase-dependent apoptosis and activates p53 in retinal ganglion cells (RGC-5).. Exp Eye Res.

[52] Iizuka Y, Hong S, Kim CY, Kim SK, Seong GJ (2008). Agmatine pretreatment protects retinal ganglion cells (RGC-5 cell line) from oxidative stress in vitro.. Biocell.

[53] Williams D, Norman G, Khoury C, Metcalfe N, Briard J, Laporte A, Sheibani S, Portt L, Mandato CA, Greenwood MT (2011). Evidence for a second messenger function of dUTP during Bax mediated apoptosis of yeast and mammalian cells.. Biochim Biophys Acta.

[54] Wang F, Zhai H, Huang L, Li H, Xu Y, Qiao X, Sun S, Wu Y (2012). Aspirin Protects Dopaminergic neurons against lipopolysaccharide-induced neurotoxicity in primary midbrain cultures.. J Mol Neurosci.

[55] Patel VP, Chu CT (2011). Nuclear transport, oxidative stress, and neurodegeneration.. Int J Clin Exp Pathol.

[56] Kaur H, Chauhan S, Sandhir R (2011). Protective effect of lycopene on oxidative stress and cognitive decline in rotenone induced model of Parkinson's disease.. Neurochem Res.

[57] Allaman I, Gavillet M, Belanger M, Laroche T, Viertl D, Lashuel HA, Magistretti PJ (2010). Amyloid-beta aggregates cause alterations of astrocytic metabolic phenotype: impact on neuronal viability.. J Neurosci.

[58] Fitzgerald M, Bartlett CA, Payne SC, Hart NS, Rodger J, Harvey AR, Dunlop SA (2010). Near infrared light reduces oxidative stress and preserves function in CNS tissue vulnerable to secondary degeneration following partial transection of the optic nerve.. J Neurotrauma.

[59] Giacco F, Brownlee M (2010). Oxidative stress and diabetic complications.. Circ Res.

[60] Del RR, Moya EA, Iturriaga R (2011). Differential expression of pro-inflammatory cytokines, endothelin-1 and nitric oxide synthases in the rat carotid body exposed to intermittent hypoxia.. Brain Res.

[61] Mozaffarieh M, Grieshaber MC, Orgul S, Flammer J (2008). The potential value of natural antioxidative treatment in glaucoma.. Surv Ophthalmol.

[62] Kong GY, Van Bergen NJ, Trounce IA, Crowston JG (2009). Mitochondrial dysfunction and glaucoma.. J Glaucoma.

[63] Osborne NN (2008). Pathogenesis of ganglion “cell death” in glaucoma and neuroprotection: focus on ganglion cell axonal mitochondria.. Prog Brain Res.

[64] Zhang J, Fu S, Liu S, Mao T, Xiu R (2000). The therapeutic effect of Ginkgo biloba extract in SHR rats and its possible mechanisms based on cerebral microvascular flow and vasomotion.. Clin Hemorheol Microcirc.

[65] Flammer J (2001). Glaucomatous optic neuropathy: a reperfusion injury.. Klin Monatsbl Augenheilkd.

[66] Szabo ME, Droy-Lefaix MT, Doly M (1997). Direct measurement of free radicals in ischemic/reperfused diabetic rat retina.. Clin Neurosci.

[67] Hao Y, Sun Y, Xu C, Jiang X, Sun H, Wu Q, Yan C, Gu S (2009). Improvement of contractile function in isolated cardiomyocytes from ischemia-reperfusion rats by ginkgolide B pretreatment.. J Cardiovasc Pharmacol.

[68] Uhler TA, Piltz-Seymour J (2008). Optic disc hemorrhages in glaucoma and ocular hypertension: implications and recommendations.. Curr Opin Ophthalmol.

[69] Bengtsson B, Leske MC, Yang Z, Heijl A (2008). Disc hemorrhages and treatment in the early manifest glaucoma trial.. Ophthalmology.

[70] Kotil K, Uyar R, Bilge T, Ton T, Kucukhuseyin C, Koldas M, Atay F (2008). Investigation of the dose-dependent antivasospasmic effect of Ginkgo biloba extract (EGb 761) in experimental subarachnoid hemorrhage.. J Clin Neurosci.

[71] Hirooka K, Tokuda M, Miyamoto O, Itano T, Baba T, Shiraga F (2004). The Ginkgo biloba extract (EGb 761) provides a neuroprotective effect on retinal ganglion cells in a rat model of chronic glaucoma.. Curr Eye Res.

[72] Ritch R (2005). Complementary therapy for the treatment of glaucoma: a perspective.. Ophthalmol Clin North Am.

[73] Ritch R (2000). Potential role for Ginkgo biloba extract in the treatment of glaucoma.. Med Hypotheses.

[74] Galassi F, Sodi A, Ucci F, Renieri G, Pieri B, Baccini M (2003). Ocular hemodynamics and glaucoma prognosis: a color Doppler imaging study.. Arch Ophthalmol.

[75] Schumann J, Orgul S, Gugleta K, Dubler B, Flammer J (2000). Interocular difference in progression of glaucoma correlates with interocular differences in retrobulbar circulation.. Am J Ophthalmol.

[76] Satilmis M, Orgul S, Doubler B, Flammer J (2003). Rate of progression of glaucoma correlates with retrobulbar circulation and intraocular pressure.. Am J Ophthalmol.

[77] Chung HS, Harris A, Kristinsson JK, Ciulla TA, Kagemann C, Ritch R (1999). Ginkgo biloba extract increases ocular blood flow velocity.. J Ocul Pharmacol Ther.

[78] Mozaffarieh M, Flammer J (2007). Is there more to glaucoma treatment than lowering IOP?. Surv Ophthalmol.

[79] Flammer J, Drance SM (1983). Effect of acetazolamide on the differential threshold.. Arch Ophthalmol.

[80] Flammer J, Drance SM (1983). Reversibility of a glaucomatous visual field defect after acetazolamide therapy.. Can J Ophthalmol.

[81] Gaspar AZ, Flammer J, Hendrickson P (1994). Influence of nifedipine on the visual fields of patients with optic-nerve-head diseases.. Eur J Ophthalmol.

[82] Quaranta L, Bettelli S, Uva MG, Semeraro F, Turano R, Gandolfo E (2003). Effect of Ginkgo biloba extract on preexisting visual field damage in normal tension glaucoma.. Ophthalmology.

[83] DeKosky ST, Fitzpatrick A, Ives DG, Saxton J, Williamson J, Lopez OL, Burke G, Fried L, Kuller LH, Robbins J, Tracy R, Woolard N, Dunn L, Kronmal R, Nahin R, Furberg C (2006). The Ginkgo Evaluation of Memory (GEM) study: design and baseline data of a randomized trial of Ginkgo biloba extract in prevention of dementia.. Contemp Clin Trials.

[84] Le Bars PL, Katz MM, Berman N, Itil TM, Freedman AM, Schatzberg AF (1997). A placebo-controlled, double-blind, randomized trial of an extract of Ginkgo biloba for dementia. North American EGb Study Group.. JAMA.

[85] Taillandier J, Ammar A, Rabourdin JP, Ribeyre JP, Pichon J, Niddam S, Pierart H (1986). Treatment of cerebral aging disorders with Ginkgo biloba extract. A longitudinal multicenter double-blind drug vs. placebo study.. Presse Med.

[86] McKenna DJ, Jones K, Hughes K (2001). Efficacy, safety, and use of ginkgo biloba in clinical and preclinical applications.. Altern Ther Health Med.

[87] Bent S, Goldberg H, Padula A, Avins AL (2005). Spontaneous bleeding associated with ginkgo biloba: a case report and systematic review of the literature: a case report and systematic review of the literature.. J Gen Intern Med.

[88] Köhler S, Funk P, Kieser M (2004). Influence of a 7-day treatment with Ginkgo biloba special extract EGb 761 on bleeding time and coagulation: a randomized, placebo-controlled, double-blind study in healthy volunteers.. Blood Coagul Fibrinolysis.

[89] Bal Dit Sollier C, Caplain H, Drouet L (2003). No alteration in platelet function or coagulation induced by EGb761 in a controlled study.. Clin Lab Haematol.

[90] DeKosky ST, Williamson JD, Fitzpatrick AL, Kronmal RA, Ives DG, Saxton JA, Lopez OL, Burke G, Carlson MC, Fried LP, Kuller LH, Robbins JA, Tracy RP, Woolard NF, Dunn L, Snitz BE, Nahin RL, Furberg CD (2008). Ginkgo biloba for prevention of dementia: a randomized controlled trial.. JAMA.

[91] Dodge HH, Zitzelberger T, Oken BS, Howieson D, Kaye J (2008). A randomized placebo-controlled trial of Ginkgo biloba for the prevention of cognitive decline.. Neurology.

[92] Brinker F (2005). July, 2005 edited excerpts from: complex herbs-complete medicines, Eclectic Medical Publications, Sandy, OR, 2004.. J Herb Pharmacother.

[93] Wolf HR (2006). Does Ginkgo biloba special extract EGb 761 provide additional effects on coagulation and bleeding when added to acetylsalicylic acid 500 mg daily?. Drugs R D.

[94] Engelsen J, Nielsen JD, Winther K (2002). Effect of coenzyme Q10 and Ginkgo biloba on warfarin dosage in stable, long-term warfarin treated outpatients. A randomised, double blind, placebo-crossover trial.. Thromb Haemost.

[95] Jiang X, Blair EY, McLachlan AJ (2006). Investigation of the effects of herbal medicines on warfarin response in healthy subjects: a population pharmacokinetic-pharmacodynamic modeling approach.. J Clin Pharmacol.

